# RNA 2*’*-O-Methyltransferase Fibrillarin Facilitates Virus Entry Into Macrophages Through Inhibiting Type I Interferon Response

**DOI:** 10.3389/fimmu.2022.793582

**Published:** 2022-04-07

**Authors:** Panpan Li, Yang Liu, Renjie Song, Lu Zhao, Jiang Yang, Fengjiao Lu, Xuetao Cao

**Affiliations:** ^1^ Department of Immunology, Institute of Basic Medical Sciences, Peking Union Medical College, Chinese Academy of Medical Sciences, Beijing, China; ^2^ Institute of Immunology, College of Life Sciences, Nankai University, Tianjin, China

**Keywords:** RNA 2*’*-O-methylation, fibrillarin, viral infection, type I interferon, innate immunity, macrophages

## Abstract

Type I interferons (IFN-I) play crucial roles in antiviral immune responses through inducing multiple antiviral interferon stimulated genes (ISGs). RNA modifications are emerging as critical post-transcriptional regulators of gene expression programs, which affect diverse biological processes. 2*’*-O-methylation (Nm) is one of the most common types of RNA modifications found in several kinds of RNA. However, the function and underlying mechanism of Nm modification in regulating viral infection and innate immunity are largely unknown. Here we found that 2*’*-O-methyladenosine (Am) on poly A+ RNA was increased in virus infected-macrophages. Functional screening identified RNA 2*’*-O-methyltransferase Fibrillarin (FBL) in facilitating viral infection. Down-regulation of FBL inhibited viral infection through blocking virus entry into macrophages. Furthermore, knockdown of FBL could reduce viral entry by increasing ISGs expression through IFN-I signaling. These results indicated that FBL-mediated Nm modifications of RNA may avoid the innate immune recognition, thereby maintain immune homeostasis. Once FBL is down-regulated, the decreased Nm modifications of RNA in macrophages may act as “non-self” RNA and be recognized by RNA sensor interferon induced with helicase C domain 1 (MDA5), leading to innate immune activation by inducing the expression of IFN-I and ISGs. Therefore, our finding reveals a new role of FBL and its mediated RNA Nm modifications in facilitating viral infection and inhibiting innate immune response, adding mechanistic insight to the RNA modifications in infection and immunity.

## Introduction

Innate immune response plays an essential role in host defenses against viral infection. Innate immune cells express kinds of pattern recognition receptors (PRRs) to identify pathogen associated molecular patterns (PAMPs) from the invading viruses, such as “non-self” viral RNAs and DNAs, which can activate the host innate immune response for the elimination of invading virus (1–3). The inducible IFN-I plays key role in establishing and modulating host defense against viral infection through inducing the expression of interferon stimulated genes (ISGs) *via* Janus kinase (JAK)-signal transducer and activator of transcription (STAT) signaling pathway (1, 3). In the interaction between viruses and the host, the immune cells can regulate gene expressions in response to the pathogen infection at multiple epigenetic levels, including histone modifications, DNA modifications, RNA modifications, and non-coding RNAs, etc. (4–6). Among those epigenetic modifiers, RNA modifications in regulating immunity and infection attract much attention (7), while most studies mainly focused on *N* ^6^-methyladenosine (m^6^A) (8–11). We previously revealed that m^6^A RNA modification–mediated down-regulation of the α-ketoglutarate dehydrogenase-itaconate pathway and cellular metabolism rewiring inhibit viral replication in macrophages (8). However, whether other types of RNA modifications also participate in viral infection or innate immunity remains largely unknown.

RNA 2*’*-O methylation (Nm) is one of the most common types of RNA modifications that are found in ribosomal RNAs (rRNAs), transfer RNAs, small nucleolar RNAs and also in messenger RNAs (mRNAs) (6). Nm modifications are formed in 2*’*-OH group of RNA riboses and respectively named as 2’-O-methyladenosine (Am), 2’-O-methylguanosine (Gm), 2’-O-methylcytidine (Cm) and 2’-O-methyluridine (Um). Nm endows nucleotides with greater hydrophobicity and affects RNA molecules in a variety of ways including the structure, stability and interaction of RNA, so as to regulate various cellular processes such as translation (6, 12–14). Research on the biological functions of Nm became possible until high-throughput sequencing methods of Nm residues have been developed, especially for low abundant mRNA (15, 16). The Nm modifications of mRNA 5*’*cap, precisely on the first and sometimes second cap-proximal nucleotides, are shown to serve as a “self-RNA” signal to prevent PRRs from recognizing self mRNA (17, 18). 2*’*-O-methylation sequencing (Nm-Seq) confirm that Nm modifications are present not only in the 5*’*cap of the mRNA, but also in the interior of some mRNAs (15). However, the physiological functions of Nm modifications in immune cells are still unknown.

Fibrillarin (FBL) is a 34 kDa nucleolar RNA 2*’*-O-methyltransferase and a highly conserved protein, which is located in the dense fibrillar component of the nucleolus (19). FBL mainly catalyzes Nm modifications on rRNA under the guidance of BOX C/D small nucleolar RNAs (snoRNAs) (19). Previous studies on FBL mainly focused on tumors. For instance, FBL contributes to tumorigenesis and is associated with poor survival in patients with breast cancer (20). Targeting FBL shows great potential correlation to an improved survival rate at low expression in breast cancer patients and association with p53, due to its pivotal role in ribosome biogenesis (21). Besides, FBL knockdown enhances the resistance in *C. elegans* against bacterial pathogens independent of the major innate immunity mediators (22). FBL also contributes to the long-distance transport of plant viruses in plants (23, 24). However, whether FBL-catalyzed Nm modifications regulate innate immunity is unclear.

By functional screening of eight RNA 2*’*-O-methyltransferases, in this study we found that FBL inhibits innate immune response by suppressing the expression of IFN-I and ISGs in macrophages, which can promote virus entry into macrophages to facilitate viral infection.

## Materials and Methods

### Mice and Cells

C57BL/6 mice (6-8 weeks old) were from Institute of Laboratory Animal Science, Chinese Academy of Medical Sciences (Beijing). The interferon-α/β receptor 1 (IFNAR1)-deficient (*Ifnar1* ^-/-^) mice (6-8 weeks old) were obtained from Jackson Laboratory. All mice were bred and maintained under specific-pathogen-free conditions. All animal experiments were performed according to the National Institutes of Health Guide for the Care and Use of Laboratory Animals, with the approval of the Animals Care and Use Committees of the Institute of Laboratory Animal Sciences of Chinese Academy of Medical Sciences (ACUC-A01-2021-040).

Mouse peritoneal macrophages were obtained as previously described (8, 25). The RAW264.7, A549 and HEK293T cell lines were obtained from American Type Culture Collection (ATCC) and cultured as required. We generated FBL-knockdown RAW264.7 cells by a CRISPR-Cas9 gene-editing system with short guide RNA sequence-containing plasmid targeting specific sequences in the genome (5’-GGAGGTCGAGGTCGAGGCGG-3’ and 5’-GCTGCCAGCTTGGAGCGGAA-3’). We used PCR followed by sequencing and immunoblotting to determine the knockdown efficiency. *MAVS* ^-/-^ A549 cells and *MDA5* ^-/-^ A549 cells were also generated by a CRISPR-Cas9 approach.

### Plasmids, Reagents, and Pathogens

FBL full-length sequences were obtained from mouse peritoneal macrophage cDNA and then cloned into pcDNA™4/myc-His A. Vesicular Stomatitis Virus (VSV) and herpes simplex virus type 1 (HSV-1) viruses were used as described previously (25).

Adenosine (132283), 2*’*-O-Methyladenosine (591363), Cytidine (119085), 2*’*-O-Methylcytidine (391517), Guanosine (979688), 2*’*-O-Methylguanosine hydrate (329290), Uridine (399796), 6-chloropurine riboside (455573), 2*’*-O-Methyluridine (488001) were obtained from J&K Scientific Ltd.

### Western Blot

These assays were performed as described previously (8). VSV-G (ab183497) antibody was obtained from Abcam. FBL (16021-1-AP), Beta Actin (66009-1-Ig) antibodies were from Proteintech. Myc-tag (2278S), RIG-I (3743S), STAT1 (14994S), P-STAT1 (9167S), IRF3 (4302S), P-IRF3 (4947S) antibodies were from Cell Signal Technology. GAPDH (M171-3) antibody was obtained from MBL International Corporation.

### RNA Extraction and Quantitative RT-PCR

Total RNA was extracted by TRIZOL reagent (Invitrogen) according to the manufacturer*’*s instructions. RNA was reversed-transcribed using the Reverse Transcription System from Toyobo (FSQ 301). Then cDNA was amplified by real-time PCR and analyzed as described previously (8). The primer sequences for qPCR analysis are listed in **Supplementary Table 1**.

### Transfection

RAW264.7 and A549 cells were transfected with Lipofectamine™ 3000 Transfection Reagent (L3000015, Thermo) or LipoMax DNA Transfection Reagent (32012, SUDGEN) for 48 h according to the manufacturer*’*s instructions.

### RNA Interference

Small interfering RNAs (siRNAs) were transfected into the mouse peritoneal macrophages and A549 cell lines with Lipofectamine™ RNAiMAX Transfection Reagent (13778150, Thermo) for 48 h following the manufacturer*’*s instructions. After 48h, the cells were harvested or infected with virus for corresponding hours. siRNAs were designed and synthesized by RiboBio (**Supplementary Table 2**). The efficiency of interference was determined by qPCR or Western blot.

### Construction of Inducible *Fbl* Knockout (*Fbl*-iKO) RAW 264.7 Cells

RAW264.7 cells with inducible expression of Cas9 by Cre-loxP system (iKO RAW264.7 cell) were conducted. In these cells, genome was inserted with Cas9 sequence and before cas9 sequence there was transcriptional termination sequences with LoxP sites at both ends (**Supplementary Figure 4D**). When iKO RAW264.7 cells were infected with lentivirus expressing Cyclization Recombination Enzyme (Cre), Cas9 expression was then induced by Cre. We constructed *Fbl*-iKO RAW264.7 cells which stably expressed *Fbl* sgRNA based on above iKO RAW264.7 cells. When *Fbl*-iKO cell line was infected with this lentivirus, Cas9 expression first induced and then the transient knockout of *Fbl* with *Fbl* sgRNA induced.

### ELISA

The concentrations of IFN-β and IFN-α in the supernatants were determined with VeriKine Mouse IFN Beta ELISA Kit (42400, PBL Interferon Source) and VeriKine Mouse IFN Alpha ELISA Kit (42120, PBL Interferon Source) according to the manufacturer*’*s instructions.

### Virus Binding and Entry Assays

For virus-binding assays, mouse peritoneal macrophages were transfected with the indicated siRNAs for 48 h and then infected with VSV (MOI=3) for 30min on the ice. Cells were washed 6 times with ice-cold PBS supplemented with 2% bovine serum albumin to remove unbound virions. Then cells were lysed and RNA was extracted. Bound virions were quantified as viral RNA (vRNA) levels *via* qRT–PCR. For virus-entry assays, mouse peritoneal macrophages transfected with the indicated siRNAs for 48 h and then infected with VSV (MOI=3) for 30min in 4°C. After 6 washes with ice-cold PBS and 2% BSA, pre-warmed 37°C medium supplemented with 2% FBS and 15 mM NH_4_Cl was added to cells. Cells were incubated at 37°C for 1 h to allow the virus to enter cells. Then cells were chilled on ice and incubated with 500 ng/ml proteinase K in PBS at 4 °C for 2h to remove residual plasma-membrane-bound virions. After 6 additional washes with ice-cold PBS and 2% bovine serum albumin. Then cells were lysed and RNA was extracted. And vRNA levels were quantified *via* qRT–PCR.

### Poly A+ RNA Purification

Poly A+ RNA was purified from total RNA with polyA tail purification using Dynabeads™ mRNA Purification Kit (61006, Thermo). The remaining rRNAs were further removed using NEB Next^®^ rRNA Depletion Kit (E6310L) from New England BioLabs (NEB).

### Relative Quantification of RNA Modifications by LC-HRMS

1~2ug isolated mRNA or total RNA were digested into single nucleosides by 1U nuclease P1 (N8630, Sigma) in 50 μl buffer containing 10mM ammonium acetate, pH 5.3 at 37°C for 12 h, followed by 42°C for 12 h, then mixed with 2 μl 1M ammonium bicarbonate, pH8.3, added 1U Bacterial Alkaline Phosphatase (18011015, Thermo) in a final reaction volume of 100 μl adjusted with water, and incubated at 37°C for 12 h. 100ul chloroform was added to the reaction solution, 80ul supernatant was extracted by centrifugation after vortexing, and 20ul 6-chloropurine riboside (50ug/ml) was added and mixed. High performance liquid chromatography (HPLC) was modified slightly of the published procedures (26). Briefly, the nucleosides were separated with Hypersil GOLD aQ 3-µm column (150-mm length × 2.1-mm inner diameter, pore size 120 Å, particle size 3 µm, Thermo), and then detected by Triple TOF 5600 Mass Spectrometer (AB SCIEX) or Orbitrap Fusion Tribrid Mass Spectrometer (Thermo). The column was equilibrated to 37°C with 0.1% formic acid in HPLC-grade water at a flow rate of 0.4 ml/min for at least 20 min. 10μl of the solution was injected into LC-MS. Mobile phase A was 0.1% formic acid aqueous solution and mobile phase B was 0.1% formic acid acetonitrile solution. The solvent gradient was described in **Supplementary Table 3**. The chromatographic profiles were obtained by high resolution mass spectrum with full scan mode.

### 2*’*-O-Methylation Sequencing

2*’*-O-methylation sequencing (Nm-Seq) was performed by CloudSeq Biotech Inc. (Shanghai, China) by following the published procedures with slight modification (15). Briefly, the RNA samples were fragmented at 95°C for 5 min with RNA Fragmentation Reagents (Thermo). RNA fragments were 3*’*-end repaired using Antarctic phosphatase (NEB) at 37°C for 30 min. Then, repaired RNA samples were oxidized/eliminated using 10 mM NaIO_4_ (Sigma) in 200 mM lysine-HCl buffer (pH 8.5, Sigma-Aldrich) in a total volume of 40 µl at 37°C for 30 min. The reaction was quenched by ethylene glycol, samples were further dephosphorylated by Shrimp Alkaline Phosphatase (NEB) at 37°C for 30 min. Eight cycles of oxidation-elimination-dephosphorylation were performed. A final round of oxidation/elimination reaction was performed, excluding dephosphorylation. Then, purified RNA samples were 5*’*phosphorylated by T4 polynucleotide kinase 3*’* phosphatase minus (NEB) at 37 °C for 60 min. Libraries were constructed from treated RNA fragments and untreated input fragments using NEBNext Small RNA Library Prep Set for Illumina (NEB). Sequencing was carried out on Illumina HiSeq4000 according to the manufacturer*’*s instructions.

Raw data was generated after sequencing, image analysis, base calling and quality filtering on Illumina HiSeq4000 sequencer. Firstly, Q30 was used to perform quality control. After adaptor-trimming and low quality reads removing by cutadapt (v1.9.1) software (27), high quality clean reads were generated. Then these clean reads were aligned to reference genome (mm10) using bowtie2 (v2.2.4) software (28) with end-to-end mode. Raw 2*’*-O-methylation counts and coverage counts were calculated by bedtools (v2.24) software and in-house scripts, then 2*’*-O-methylation-ratio (defined as: count/coverage) and 2*’*-O-methylation-fc (defined as: 2*’*-O-methylation-ratio/Input-2*’*-O-methylation-ratio) were also calculated. 2*’*-O-methylation sites were annotated with gene information by bedtools software. And the 2*’*-O-methylation sites were visualized in IGV (v2.64) software (29). Sequence motifs on Nm peaks were identified by HOMER (30).

### RNA High Throughput Sequencing

Briefly, total RNA was used for removing the rRNAs with NEBNext rRNA Depletion Kit (NEB) following the manufacturer*’*s instructions. RNA libraries were constructed by using NEBNext^®^ Ultra™ II Directional RNA Library Prep Kit (NEB) according to the manufacturer*’*s instructions. RNA high throughput sequencing (RNA-Seq) was performed by Cloud-Seq Biotech (Shanghai, China). Two independent biological replicates were performed for RNA-seq.

### Statistical Analysis

Data are expressed as the mean ± standard error of the mean (SEM) from at least three independent triplicated experiments. The number of individuals and repeated experiments are stated in each figure legend. All data was analyzed using the GraphPad Prism software version 8.4.2. The statistical significance of comparisons between two groups was determined with Two-tailed unpaired Student*’*s *t* test. The relative gene expression data was acquired using the 2^ΔΔCT^ method. *P*-value: ns, *P >*0.05; *, *P* ≤ 0.05; **, *P* ≤ 0.01; ***, *P* ≤ 0.001; ****, *P* ≤ 0.0001.

## Results

### Increased Am Modification on Poly A+ RNA in Macrophages Upon Viral Infection

**Figure 1:**
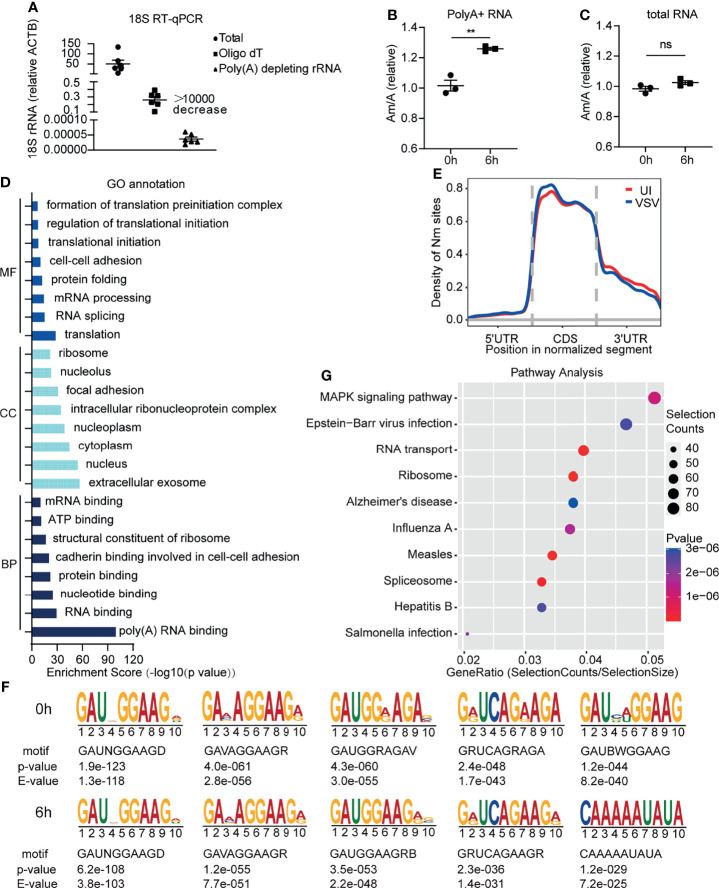
Increased Am modification on poly A+ RNA in macrophages upon viral infection. **(A)** Determination of poly A+ RNA purity through RT-qPCR with primers specific to 18S rRNA and *Actb*. Total, total RNA; Oligo dT, poly A+ purified by Oligo dT beads; Poly A+ depleting rRNA, poly A+ purified by Oligo dT beads and rRNA depleting kit (n=6); **(B)** Quantification of the Am/A ratio in poly A+ RNA of RAW264.7 cells with or without VSV infection (n=3). 0h, RNA from RAW264.7 cells; 6h, RNA from RAW264.7 cells infected with VSV (MOI=1) for 6 h; **(C)** Quantification of the Am/A ratio in total RNA of RAW264.7 cells with or without VSV infection (n=3). 0h, RNA from RAW264.7 cells; 6h, RNA from RAW264.7 cells infected with VSV (MOI=1) for 6 h; **(D)** GO terms of MF, CC, BP for Nm-methylated transcripts of RAW264.7 at the steady state; MF, Molecular function; CC, Cell Compartment; BP, Biological process; **(E)** Metagene profile of Nm sites distribution along a normalized mRNA transcript; **(F)** Sequence logo of enriched motifs behind Nm sites identified by HOMER; **(G)** KEGG pathway enrichment for Nm up-regulated genes after viral infection. UI, poly A+ RNA from RAW264.7 cells; VSV, poly A+ RNA from RAW264.7 cells infected with VSV (MOI=1) for 6 h. All data are mean ± SEM of biologically independent samples.ns, not significant, **P < 0.01, two-tailed unpaired Student*’*s *t* test.

In order to identify RNA modifications that regulate virus infection or innate immunity, we used high resolution mass spectrometry to observe the changed types of RNA modifications in RAW264.7 macrophages induced by VSV infection. To prevent rRNA modifications from interfering with the detection of low RNA modification level on mRNA, we optimized the poly A+ RNA purification method by adding a rRNA removal step, and the rRNA residue in this optimized method was much lower than the traditional purification method of Oligo dT beads **(** **Figure 1A** **)**. We found that the level of Am was increased on poly A+ RNA of RAW264.7 macrophages after VSV infection, while Am level in total RNA remained unchanged after VSV infection **(** **Figures 1B, C** and **Supplementary Figures 1A, B** **).**

Am is one type of Nm modifications. To provide functional insights into whether mRNAs carrying Nm modification in macrophages are linked to antiviral innate immunity, we performed transcriptome-wide Nm-seq on the poly A+ RNA of RAW264.7 macrophages with or without VSV infection. We identified 6808 Nm sites (fold change (FC)≥4), 5643 of which had a minimal Nm-seq count of ten reads. Next, Gene Ontology (GO) analyses of these methylated genes were performed and showed that the significantly enriched methylated genes were as follows: translation and RNA process for molecular function (MF); extracellular exosome, nucleus and cytoplasm for cell compartment (CC); RNA and nucleotide binding, protein binding for biological process (BP) (**Figure 1D** and **Supplementary Table 4**). The most dramatic BP was binding, especially binding of RNA and nucleotide, suggesting that mRNA Nm modifications might play an important role in the nucleus, including RNA splicing and processing. Most of the Nm peaks were apparently positioned in coding DNA sequence (CDS), which was consistent with the other reported Nm-seq result (15) (**Figure 1E**). An unbiased search for common motifs enriched in segments around Nm peak summits was performed. The most significantly enriched motifs were slightly changed after VSV infection (**Figure 1F**), which may be due to the differences in the relative abundance of different RNAs before and after viral infection. Interestingly, we found that the Nm modifications are changed on the mRNA of a large number of genes related to viral infection (**Figure 1G** and **Supplementary Table 5**). These results of GO and KEGG analysis suggested that Nm RNA modifications may be involved in regulating virus infection and antiviral innate immunity.

### Functional Screening Identifies RNA 2*’*-O-Methyltransferase FBL to Facilitate Viral Infection

**Figure 2:**
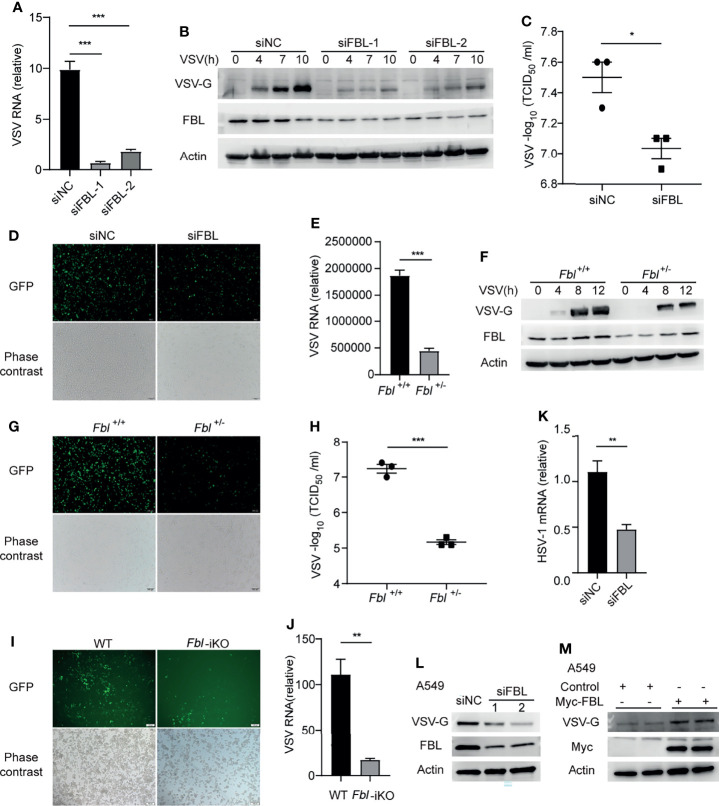
RNA 2*′*-O-methyltransferase FBL facilitates viral infection. **(A)** RT-qPCR of VSV RNA in mouse peritoneal macrophages transfected with the indicated siRNAs for 48 h and then infected with VSV (MOI=3) for 10 h (n=3); **(B)** Western blot of VSV-G protein in mouse peritoneal macrophages transfected with the indicated siRNAs for 48 h and then infected with VSV (MOI=3) for 0, 4, 7, 10 h (n=3); **(C)** VSV titers by median tissue culture infectious dose (TCID_50_) assay in supernatants of peritoneal macrophages transfected with the indicated siRNAs for 48 h and then infected with VSV for 12 h (n=3); **(D)** Fluorescence microscopy images of mouse peritoneal macrophages transfected with the indicated siRNAs for 48 h and then infected with GFP-VSV (MOI=5) for 8 h. Scale bar, 100 μm (n=3); **(E)** RT-qPCR of VSV RNA in WT and *Fbl* ^+/-^ RAW264.7 cells infected with GFP-VSV for 8 h (n=4); **(F)** Western blot of VSV-G levels in WT and *Fbl* ^+/-^ RAW264.7 cells infected with GFP-VSV for 0, 4, 8, 12 h (n=3); **(G)** Fluorescence microscopy images of WT and *Fbl* ^+/-^ RAW264.7 cells infected with GFP-VSV for 8 h. Scale bar, 100 μm (n=3); **(H)** VSV titers by TCID_50_ assay in supernatants of WT and *Fbl* ^+/-^ RAW264.7 cells infected with GFP-VSV for 12 h (n=3); **(I)** Fluorescence microscopy images of WT and *Fbl*-iKO RAW264.7 cells infected with GFP-VSV for 16 h. Scale bar, 100 μm (n=3); **(J)** RT-qPCR of VSV RNA in WT and *Fbl*-iKO RAW264.7 cells infected with GFP-VSV for 8 h (n=3); **(K)** qRT-PCR of HSV-1 RNA in mouse peritoneal macrophages transfected with the indicated siRNAs for 48 h and then infected with HSV-1 for 10 h (n=4); **(L)** Western blot of VSV-G protein in A549 cells transfected with the indicated siRNAs (siFBL-1 and siFBL-2) for 48 h and then infected with VSV (MOI=0.5) for 8 h (n=3); **(M)** Western blot of VSV-G protein in A549 cells transfected with empty vector or FBL expression vector for 48 h and then infected with VSV (MOI=0.5) for 8 h (n=3). All data are mean ± SEM of biologically independent samples. *P < 0.05, **P < 0.01, ***P < 0.001, two-tailed unpaired Student*’*s *t* test.

Then we focused on identifying which RNA 2*’*-O-methyltransferase may participate in regulating viral infection. Through performing functional screening of eight Nm associated enzymes (FTSJ1, FTSJ2, FTSJ3, FBL, CMTR1, CMTR2, MRM1, MRM3) *via* siRNAs-mediated knockdown, we found that knockdown of FBL inhibited VSV infection of mouse peritoneal macrophages, down-regulation of FTSJ1 promoted VSV infection in mouse peritoneal macrophages, and the effects of different siRNA of other enzymes were inconsistent or had no significant effects on VSV infection **(** **Figure 2A** and **Supplementary Figures 2A, B** **)**. FBL is an essential nucleolar protein that participates in pre-rRNA methylation and processing, and also is an extremely well-conserved protein during the evolution from archaea to human (19). We found that FBL was the highest expressed RNA 2*’*-O-methyltransferase in mouse peritoneal macrophages (**Supplementary Figure 3A**). We retrieved gene expression omnibus (GEO) dataset GDS4185 which contains FBL mRNA expression data of isolated CD4+ T cells and CD19+ B cells from the blood of Systemic Lupus Erythematosus (SLE) patients and healthy controls. We found that FBL expressions in CD19+ B cells and CD4+ T cells of SLE patients were lower than that of healthy controls (**Supplementary Figures 3B, C** **)**, suggesting that FBL may play an immunomodulatory role. FBL was reported that mainly catalyzes Nm modifications on rRNA under the guidance of BOX C/D snoRNAs (19). However, whether FBL regulates immune response is largely unknown. This inspired us to investigate the role of FBL in infection and innate immunity.

Furthermore, we observed that siRNA-mediated knockdown of FBL decreased VSV protein expression and VSV titers in cell supernatant **(** **Figures 2B–D** **)**. Because FBL deficiency induces lethality (31), FBL knockout monoclonal cell lines and *Fbl^-/-^* mice cannot be obtained. We generated FBL-knockdown RAW264.7 cells by CRISPR-Cas9 gene-editing systems **(** **Supplementary Figures 4A–C** **)**, and these *Fbl* ^+/-^ RAW264.7 cells also showed decreased intracellular virus production upon infection of recombinant green fluorescent protein-expressing VSV (GFP-VSV) **(** **Figures 2E–H** **)**. We further verified that FBL promoted VSV infection by *Fbl*-iKO RAW264.7 cells **(** **Figures 2I**, **J** **;** **Supplementary Figures 4D–F** **)**. Besides, knockdown of FBL significantly inhibited the infection of DNA virus HSV-1 in mouse peritoneal macrophages **(** **Figure 2K** **)**, in addition to RNA virus VSV. Consistently, knockdown of FBL inhibited VSV infection while overexpression of FBL facilitated VSV infection in human A549 cells **(** **Figures 2L, M** **)**. These results demonstrate that FBL facilitates viral infection.

### FBL Facilitates VSV Entry Into Macrophages at the Early Stage of Infection

**Figure 3:**
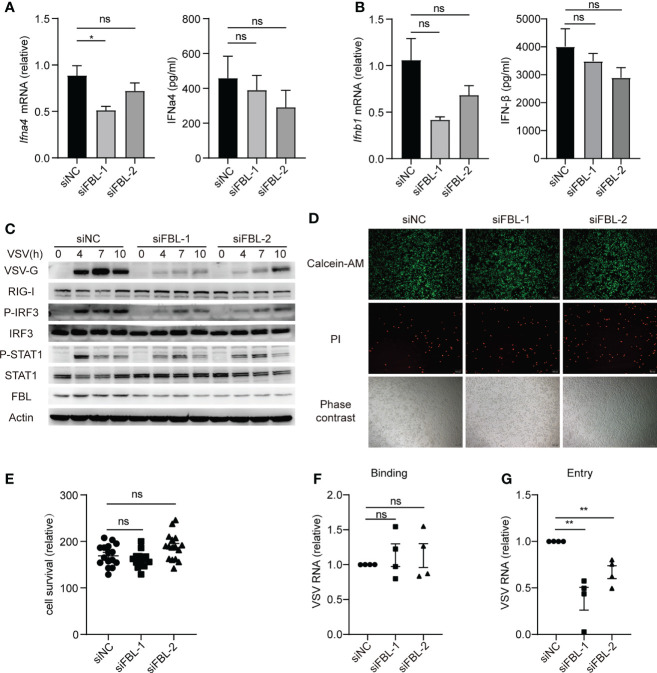
FBL facilitates VSV entry into macrophages at the early stage of infection. **(A)** qRT-PCR of *Ifnα4* mRNA and ELISA of IFNα4 protein level in mouse peritoneal macrophages transfected with the indicated siRNAs for 48 h and then infected with VSV for 10 h (n=3); **(B)** qRT-PCR of *Ifnb1* mRNA and ELISA of IFNβ protein level in mouse peritoneal macrophages transfected with the indicated siRNAs for 48 h and then infected with VSV for 10 h (n=3); **(C)** Western blot of innate signaling activation in mouse peritoneal macrophages transfected with the indicated siRNAs for 48 h and then infected with VSV for 0, 4, 7, 10 h; **(D)** Mouse peritoneal macrophages were transfected with the indicated siRNAs for 48 h then to test cell activity and cytotoxicity by fluorescence microscopy images. Scale bar, 100 μm (n=4); **(E)** Calcein/PI cell activity and cytotoxicity assay kit to test cell survival (n=4, repeated four times); **(F)** Mouse peritoneal macrophages transfected with the indicated siRNAs for 48 h and then infected with VSV for 30 min on the ice, then bound virions were quantified as viral RNA (vRNA) levels *via* qRT-PCR (n=4); **(G)** Mouse peritoneal macrophages transfected with the indicated siRNAs for 48 h and then infected with VSV for 30min in 4°C (n=4). After removal of unbound virus, the temperature was increased to 37°C for 1 h to allow internalization. Then quantify vRNA levels *via* qRT–PCR. Data are mean ± SEM of biologically independent samples. ns, not significant, *P < 0.05, **P < 0.01, two-tailed unpaired Student*’*s *t* test.

IFN-I and ISGs play important roles in antiviral innate immunity. However, the mRNA and protein expressions of IFN-α and IFN-β, as well as the activation of IFN-I signaling pathway in FBL-knockdown mouse peritoneal macrophages were not increased than that in the control cells during VSV infection **(** **Figures 3A–C** **)**. Besides, we found that FBL knockdown did not affect the viability of mouse peritoneal macrophages **(** **Figures 3D, E** **)**. FBL knockdown significantly inhibited the VSV protein expression in the early stage of infection **(** **Figure 3C** **)**. Therefore, we hypothesize that FBL may affect the early stages of the VSV life cycle. To test this idea, we performed VSV binding and entry assays in mouse peritoneal macrophages and found that FBL knockdown did not affect VSV binding, but inhibited VSV entry into mouse peritoneal macrophages **(** **Figures 3F, G** **)**.

### FBL Inhibits the Expression of ISGs in Macrophages Under Steady State

**Figure 4:**
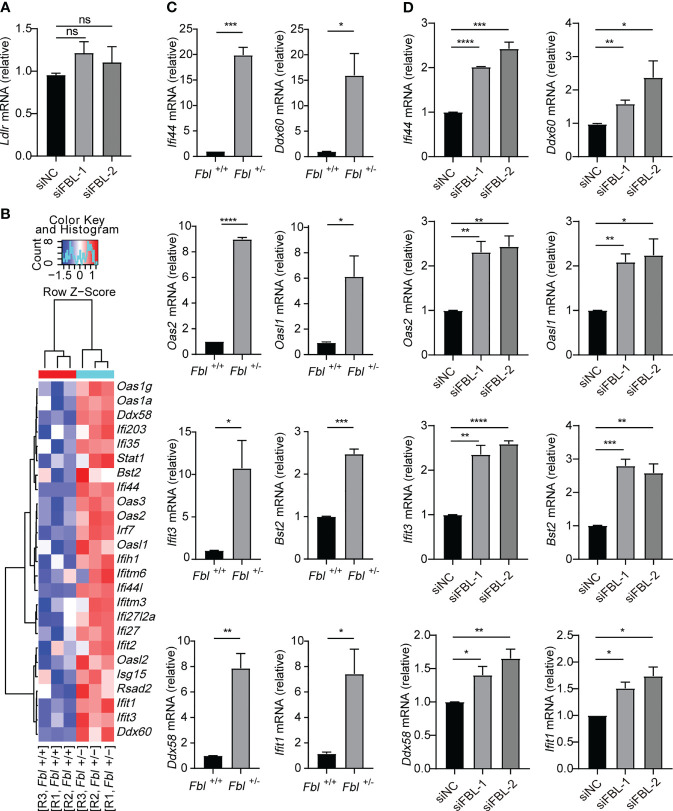
FBL inhibits the expression of ISGs in macrophages. **(A)** qRT-PCR analysis of *Ldlr* mRNA in mouse peritoneal macrophages transfected with the indicated siRNAs for 48 h (n=3); **(B)** Hotmap showing normalized transcript expression levels of immune-associated genes in WT and *Fbl* ^+/-^ RAW264.7 cells (n=3); **(C)** qRT-PCR analysis of *Ifi44*, *Oas2*, *Ifit3*, *Ddx58*, *Ddx60*, *Oasl1*, *Bst2*, *Ifit1* mRNA in WT and *Fbl* ^+/-^ RAW264.7 cells (n=3); **(D)** qRT-PCR analysis of *Ifi44*, *Oas2*, *Ifit3*, *Ddx58*, *Ddx60*, *Oasl1*, *Bst2*, *Ifit1* mRNA in mouse peritoneal macrophages transfected with the indicated siRNAs for 48 h (n=3). Data are mean ± SEM of biologically independent samples. ns, not significant, *P<0.05, **P<0.01, ***P<0.001, ****P<0.0001, two-tailed unpaired Student*’*s *t* test.

It was reported that low density lipoprotein receptor (LDLR) and other members of this receptor family serve as VSV receptors on host cells (32, 33). We found that knockdown of FBL did not influence the expression of LDLR **(** **Figure 4A** **)**. Therefore, FBL-promoted VSV entry into macrophages in the early stage of VSV infection was not due to the regulation of LDLR expression in macrophages.

In order to investigate the underlying mechanism of FBL in facilitating VSV entry into macrophages, we performed RNA-seq and found that mRNA expressions of many ISGs were up-regulated in *Fbl* ^+/-^ RAW264.7 cells at the steady state without viral infection **(** **Figure 4B** and **Supplementary Table 6** **)**. Quantitative reverse transcription PCR (qRT-PCR) analysis also confirmed that the mRNA expressions of *Ifi44*, *Oas2*, *Ifit3*, *Ddx58*, *Ddx60*, *Oasl1*, *Bst2*, *Ifit1* were up-regulated in *Fbl* ^+/-^ RAW264.7 cells **(** **Figure 4C** **)**. Besides, siRNA-mediated knockdown of FBL in mouse peritoneal macrophages also increased the expressions of above ISGs at the steady state **(** **Figure 4D** **)**.

Therefore, FBL may act as an immunosuppressive factor under physiological conditions. FBL knockdown leads to increased expression of antiviral immune genes, thus inhibiting VSV entry into the “antiviral primed-macrophages”.

### Knockdown of FBL Reduces Viral Entry by Increasing ISGs Expression Through IFN-I Signaling

**Figure 5:**
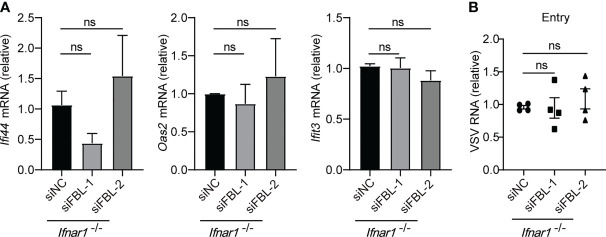
Knockdown of FBL reduces viral entry by increasing ISGs expression through IFN-I signaling. **(A)** qRT-PCR analysis of *Ifi44*, *Oas2*, *Ifit3* mRNA in *Ifnar1^-/-^* mouse peritoneal macrophages transfected with the indicated siRNAs for 48 h (n=3); **(B)** VSV RNA levels in *Ifnar1^-/-^* peritoneal macrophages transfected with the indicated siRNAs for 48 h and then infected with VSV for 30 min in 4°C. After removal of unbound virus, the temperature was increased to 37°C to allow internalization. Then quantify VSV RNA levels *via* qRT-PCR (n=4). Data are mean ± SEM of biologically independent samples. ns, not significant, two-tailed unpaired Student*’*s *t* test.

IFN-I induces the expression of ISGs through interferon-α/β receptor (IFNAR)-JAK-STAT signaling pathway (1, 3). Whether FBL regulates these antiviral immune genes through IFN-I signaling? In *Ifnar1* ^-/-^ mouse macrophages, knockdown of FBL could not promote ISGs expression **(** **Figure 5A** **)**. Meanwhile, we found that FBL knockdown did not affect VSV entry into *Ifnar1^-/-^* mouse peritoneal macrophages anymore **(** **Figure 5B** **)**. Besides, down-regulation of FBL did not regulate the expression of ISGs directly in macrophages when stimulated with enough IFN-β **(** **Supplementary Figures 5A, B** **)**. Thus, FBL facilitates VSV entry into macrophages depending on the impairment of IFN-I signaling pathway.

### FBL-Mediated RNA 2*’*-O Methylation Suppresses Innate Immune Activation and IFN-I Expression

**Figure 6:**
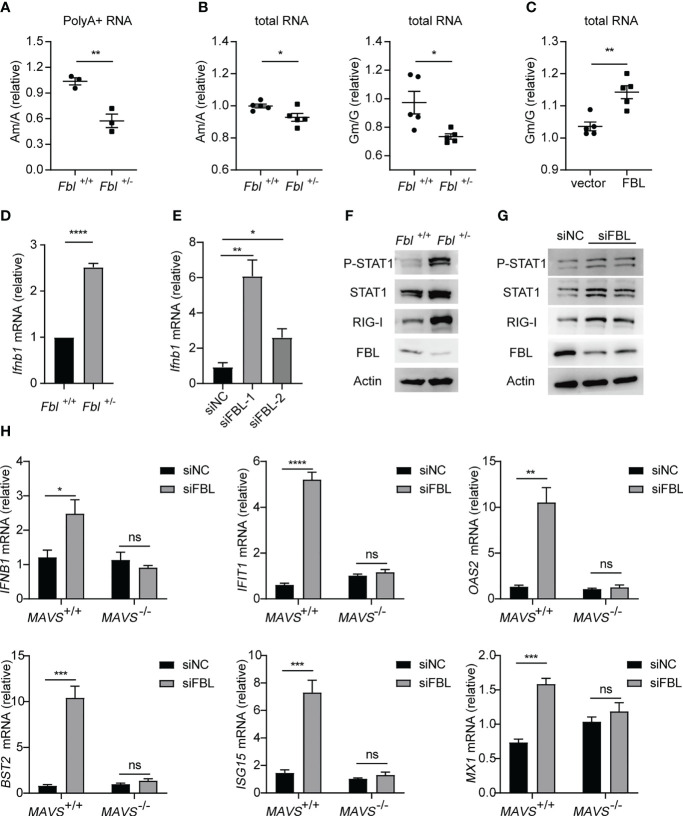
FBL-mediated RNA 2’-O methylation suppresses IFN-I expression and signaling in macrophages. **(A)** Quantification of the Am/A ratio in poly A+ RNA of WT and *Fbl* ^+/-^ RAW264.7 cells (n=3); **(B)** Quantification of the Am/A and Gm/G ratio in total RNA of WT and *Fbl* ^+/-^ RAW264.7 cells (n=5); **(C)** Quantification of the Am/A, Cm/C, Gm/G ratio in total RNA of *Fbl* ^+/-^ RAW264.7 cells transfected with empty vector and vector encoding mouse FBL for 30 h (n=5); **(D)** qRT-PCR analysis of *Ifnb1* mRNA in WT and *Fbl* ^+/-^ RAW264.7 cells (n=3); **(E)** qRT-PCR analysis of *Ifnb1* mRNA in mouse peritoneal macrophages transfected with the indicated siRNAs for 48 h (n=3); **(F)** Western blot of interferon signaling in WT and *Fbl* ^+/-^ RAW264.7 cells; **(G)** Western blot of interferon signaling in mouse peritoneal macrophages transfected with the indicated siRNAs for 48 h; **(H)** qRT-PCR analysis of *IFNB1*, *IFIT1*, *OAS2*, *BST2*, *ISG15*, *MX1* mRNA in *MAVS* ^+/+^ and *MAVS* ^-/-^ A549 cells transfected with the indicated siRNAs for 48 h (n=4). All data are mean ± SEM of biologically independent samples. ns, not significant, *P<0.05, **P<0.01, ***P < 0.001, ****P<0.0001, two-tailed unpaired Student*’*s *t* test.

FBL usually catalyzes the formation of Nm modifications on rRNA under the guidance of BOX C/D snoRNAs (6). A recent study reported that FBL and two box C/D snoRNAs (U51 and U32A) lead to Nm modification in the protein-coding region of peroxidasin (Pxdn) mRNA (13). This enlightened us that FBL may facilitate VSV entry by catalyzing Nm modification on poly A+ mRNA and rRNA broadly. We further proved this hypothesis by finding that the levels of Am modification were decreased on poly A+ RNA of *Fbl* ^+/-^ RAW264.7 macrophages compared to wide type cells **(** **Figure 6A** **)**. As rRNA is the most abundant type of RNA (80 to 85% of total RNA). We also measured Nm modifications in total RNA, and found that the levels of Am, Gm were decreased on total RNA of *Fbl* ^+/-^ RAW264.7 macrophages compared to wide type macrophages **(** **Figure 6B** **)**. While overexpression of mouse FBL led to increased Nm levels in *Fbl* ^+/-^ RAW264.7 cells **(** **Figure 6C** **)**. These results showed that FBL catalyzes the formation of Nm modifications directly.

Nm modification of capped mRNA has been reported as a molecular signature for the distinction of self and nonself mRNA by RNA sensor MDA5 (17, 18). Specifically, West Nile virus mutant (E218A) that lacks 2*’*-O-methyltransferase activity was recognized and attenuated in wild-type primary cells and mice, but was pathogenic in the absence of IFN-I signaling (17). The induction of IFN-I by coronavirus mutants lacking 2*’*-O-methyltransferase was dependent on the cytoplasmic RNA sensor MDA5 (18). Based on the relationship between FBL and Nm, we speculated that the lower Nm modification levels on host self RNA of the FBL knockdown macrophages could be recognized as “non-self” RNA by PRRs, so as to activate the innate immune response and subsequently amplify the downstream signals of IFN-I in macrophages. Then, our results showed that both the mRNA expressions of IFN-β and anti-viral immune signaling activation were increased in FBL knockdown macrophages (**Figures 6D–G**). Besides, in *MAVS*-deficient A549 cells, down-regulation of FBL did not increase the expression of IFN-I and ISGs compared with that in wild-type A549 cells (**Figure 6H**). These imply that the sensor upstream of MAVS might initiate the IFN-I signal in FBL-deficient cells.

### FBL Deficiency Increases IFN-I Signaling and ISGs Expression Through RNA Sensor MDA5

**Figure 7:**
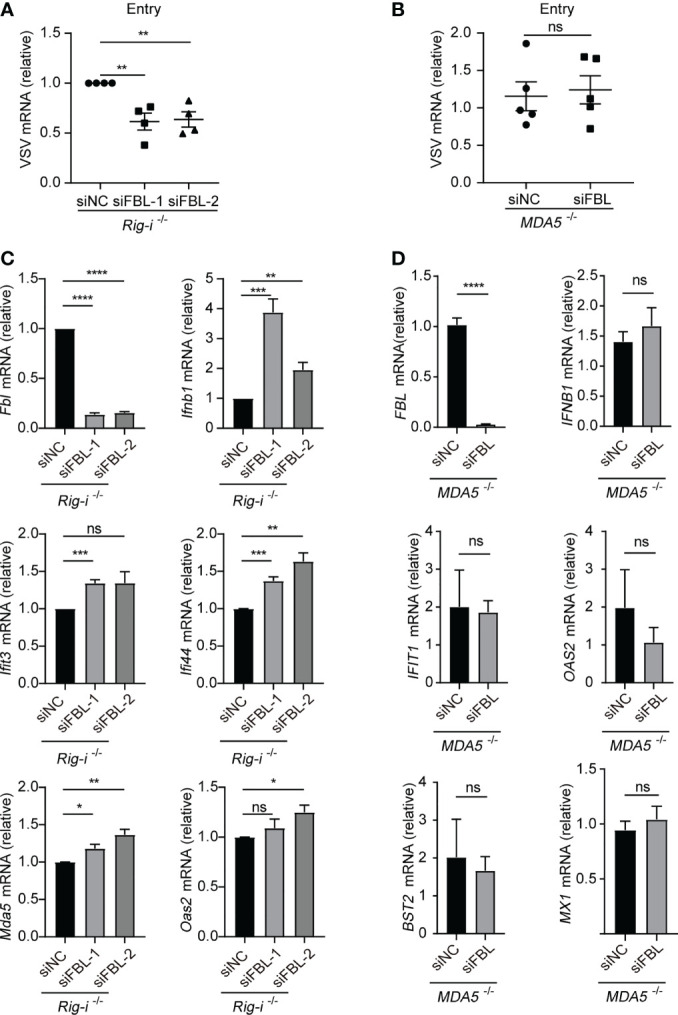
FBL deficiency increases IFN-I signaling and ISGs expression *via* RNA sensor MDA5 **(A)** VSV RNA levels in *Rig-i^-/-^* mouse peritoneal macrophages transfected with the indicated siRNAs for 48 h and then infected with VSV for 30 min in 4°C. After removal of unbound virus, the temperature was increased to 37 °C to allow internalization. Then quantify VSV RNA levels *via* qRT-PCR (n=4); **(B)** VSV RNA levels in *MDA5^-/-^* A549 cells transfected with the indicated siRNAs for 48 h and then infected with VSV for 30 min in 4°C. After removal of unbound virus, the temperature was increased to 37 °C to allow internalization. Then quantify VSV RNA levels *via* qRT-PCR (n=5); **(C)** qRT-PCR analysis of *Fbl*, *Ifnb1*, *Ifit3*, *Ifi44*, *Mda5*, *Oas2* mRNA in *Rig-i^-/-^* mouse peritoneal macrophages transfected with the indicated siRNAs for 48 h (n=3); **(D)** qRT-PCR analysis of *FBL*, *IFNB1*, *IFIT1*, *OAS2*, *BST2*, *MX1* mRNA in *MDA5* ^-/-^ A549 cells transfected with the indicated siRNAs for 48 h (n=6). Data are mean ± SEM of biologically independent samples. ns, not significant, *P<0.05, **P<0.01, ***P<0.001, ****P<0.0001, two-tailed unpaired Student*’*s *t* test.

Based on the blocking effect of MAVS in FBL-mediated suppression of IFN-I signaling and ISGs expression, we next explored the RNA sensor RIG-I and MDA5, respectively. We found that down-regulation of FBL still inhibited VSV entry into *Rig-i^-/-^* mouse peritoneal macrophages **(** **Figure 7A** **)**. While, knockdown of FBL did not affect VSV entry process in *MDA5* ^-/-^ A549 cells **(** **Figure 7B** **)**. Besides, knockdown of FBL could not inhibit the expression of ISGs any longer when loss of MDA5 rather than RIG-I under steady state **(** **Figures 7C, D** **)**.

**Figure 8:**
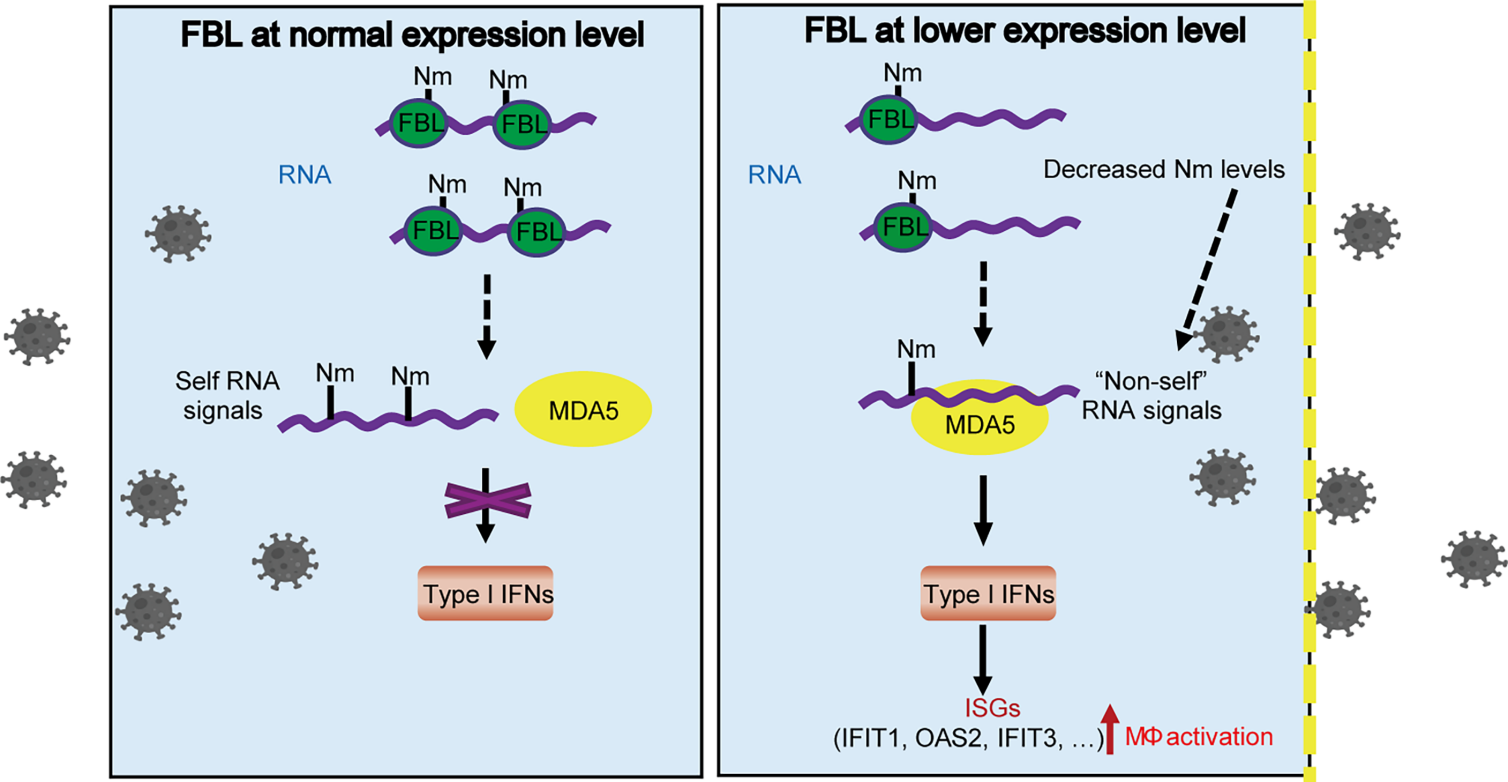
A proposed model for FBL-mediated RNA 2*’*-O methylation in promoting viral entry into macrophages by inhibiting MDA5-mediated IFN-I and ISGs expression. FBL directly catalyzes the formation of Nm RNA modifications. When FBL is expressed at a low level, the RNA with decreased Nm modification levels may be recognized as “non-self” RNAs by MDA5, which promotes the expression of IFN-I at the steady state, and then induces the expression of antiviral ISGs, such as IFIT1, OAS2, IFIT3 and so on. This “Primed immune activated state” in macrophages, upon FBL is inhibited in response to viral infection, contributes to blockade of the viral entry.

Taken together, our results indicate that FBL directly catalyzes the formation of Nm RNA modifications. Lower expression of FBL leads to decreased Nm modification levels on host RNA, which may be recognized as “non-self” RNAs by MDA5, thus promoting the expression of IFN-I at the steady state, and then induces the expression of antiviral ISGs, such as IFIT1, OAS2, IFIT3 and so on. This “Primed immune activated state” in macrophages, upon FBL is inhibited during viral infection, contributes to blockade of the viral entry **(** **Figure 8** **)**.

## Discussion

Viruses use host factors to complete the life cycles. Multiple host proteins inhibit the viral infection process by targeting different stages of the viral life cycle (34). RNA modifications regulate gene expressions to effect immunity and infection (7). We previously revealed that m^6^A RNA modification inhibits viral replication through down-regulating the α-ketoglutarate dehydrogenase-itaconate pathway and reprogramming cellular metabolism (8). Here we found that Nm RNA modification and RNA 2’-O-methyltransferase FBL also regulate viral infection.

FBL affects ribosome heterogeneity by regulating ribose methylation of rRNA to regulate translation, and plays an important role in the process of cell proliferation, senescence, tumor genesis and development (35). It has been reported that FBL regulates bacterial infection, independent of the p38 MAPK pathway, autophagy, or ubiquitin-proteasome (22). FBL is related to human diseases. The GEO dataset GDS4185 shows that FBL expressions in CD19+ B cells and CD4+ T cells of SLE patients are lower than that of healthy controls. Besides, FBL is highly expressed in a variety of tumors and has potential as a therapeutic target for tumors (21). Whether the abnormal expression of FBL in SLE and various tumors related to its immunosuppressive function need further investigations.

We wonder which process of VSV life cycle (that is binding, entry, uncoating, biosynthesis, assembly maturation and release) that FBL takes effect. In the VSV entry assay (**Figure 3G**), the low-temperature conditions (VSV could not undergo the normal process of life cycle) inhibited the VSV’s life cycle and only allowed VSV to enter mouse peritoneal macrophages slowly. However, under normal culture condition (37°C) (**Figure 2**), when VSV infected mouse macrophages, VSV could rapidly proliferate in the cells. Firstly, VSV binding analysis proved that FBL did not affect VSV binding to the macrophages. Secondly, the entry experiment showed that FBL affected VSV entry process. Therefore, fewer viruses can enter FBL-knockdown macrophages in the initial stage of infection.

In FBL-deficient immune cells, which PRR misidentifies self RNA with reduced Nm modifications as “non-self” RNA to activate the immune signaling pathway still needs further investigations. For RNA-sensing in the cytoplasm, RIG-I monitors the uncapped 5’-ends of RNA molecules (36). The carboxy-terminal domain (CTD) has a pocket binding specifically to 5’-PPP or 5’-PP groups and also contacts the unmethylated 2’-O group of the first nucleotide (36). Besides, 5’-Capped mRNA with Nm modifications could not be sensed by MDA5 (17, 18). In *MAVS*-deficient A549 cells, down-regulation of FBL cannot increase the expression of IFN-I and ISGs, implying that the RNA sensor upstream of MAVS might account for the activation of IFN-I signal in FBL-deficient macrophages. Our results further showed that MDA5 is the RNA sensor responsible for the activation of IFN signal induced by FBL deficiency.

Our results reveal that FBL inhibits the expression of IFN-I and ISGs by suppressing the innate immune activation, which promotes virus entry and further viral infection in macrophages. In sum, we propose the following working model of FBL in viral infection and immunity. FBL directly catalyzes the formation of Nm RNA modifications. When FBL is at low expression level, the reduced Nm modification may be recognized as “non-self” RNA by MDA5, which activates innate immune response and promotes the IFN-I expression thus widely increasing the expression of antiviral ISGs, such as IFIT1, OAS2, IFIT3 and so on. Our findings also indicate the possible role of FBL in homeostasis maintenance by preventing autoinflammation. This study may provide a potential target for the control of infectious diseases and autoimmune diseases.

## Data Availability Statement

The datasets presented in this study can be found in online repositories. The names of the repository/repositories and accession number(s) can be found below: https://www.ncbi.nlm.nih.gov/geo/, GSE185660 and https://www.ncbi.nlm.nih.gov/geo/, GSE185661.

## Ethics Statement

The animal study was reviewed and approved by the Animals Care and Use Committees of the Institute of Laboratory Animal Sciences of Chinese Academy of Medical Sciences (ACUC-A01-2021-040).

## Author Contributions

XC designed the experimental approach and supervised the study. PL, YL, RS, LZ, JY, and FL performed experiments. PL, YL, and XC analyzed data and wrote the paper. All authors contributed to the article and approved the submitted version.

## Funding

This work was supported by grants from the National Natural Science Foundation of China (81788101, 82071793), and the Chinese Academy of Medical Sciences Innovation Fund for Medical Sciences (2021-I2M-1-017).

## Conflict of Interest

The authors declare that the research was conducted in the absence of any commercial or financial relationships that could be construed as a potential conflict of interest.

## Publisher’s Note

All claims expressed in this article are solely those of the authors and do not necessarily represent those of their affiliated organizations, or those of the publisher, the editors and the reviewers. Any product that may be evaluated in this article, or claim that may be made by its manufacturer, is not guaranteed or endorsed by the publisher.

## Supplementary Material

Supplementary Figure 1 Increased Am modification levls on poly A+ RNA in macrophages upon viral infection **(A)** Quantification of the Am/G, Am/C and Am/U ratio in poly A+ RNA of RAW264.7 cells with or without VSV infection (n=3); **(B)** Quantification of the Am/G, Am/C and Am/U ratio in total RNA of RAW264.7 cells with or without VSV infection (n=3). 0h, RNA from RAW264.7 cells; 6h, RNA from RAW264.7 cells infected with VSV (MOI=1) for 6 h. All data are mean ± SEM of biologically independent samples. ns, not significant, **P*<0.05, ***P*<0.01, two-tailed unpaired Student's t test.

Supplementary Figure 2 RNAi screening of RNA 2′-O-methyltransferases in regulation of viral infection in macrophages **(A)** qRT-PCR of VSV RNA in mouse peritoneal macrophages transfected with the corresponding siRNA for 48h then infected with VSV (MOI=3) for 10 h (n=3). **(B)** qRT-PCR analysis mRNA expressions of *Ftsj1*, *Ftsj2*, *Ftsj3*, *Fbl*, *Cmtr1*, *Cmtr2*, *Mrm1*, *Mrm3* in mouse peritoneal macrophages transfected with the corresponding siRNA for 48 h (n =3). All data are mean ± SEM of biologically independent samples. ***P*<0.01, ****P*<0.001, *****P*<0.0001, two-tailed unpaired Student’s t test.

Supplementary Figure 3 Expression of FBL in mouse macrophages and human disease **(A)** qRT-PCR analysis mRNA expressions of *Ftsj1*, *Ftsj2*, *Ftsj3*, *Fbl*, *Cmtr1*, *Cmtr2*, *Mrm1*, *Mrm3* in mouse peritoneal macrophages under steady state (n=3). Data are mean ± SEM of biologically independent samples; **(B)** FBL and FTSJ1 expression levels in CD19+ B cells of SLE patients (n=9) and healthy controls(n=14). Data are mean ± SEM resourced from GEO dataset GDS4185; **(C)** FBL and FTSJ1 expression levels in CD4+ T cells of SLE patients (n=9) and healthy controls (n=14). Data are mean ± SEM resourced from GEO dataset GDS4185. ns, not significant, **P*<0.05, ***P*<0.01, two-tailed unpaired Student's t test.

Supplementary Figure 4 RNA 2′-O-methyltransferase FBL facilitates viral infection **(A)** CRISPR/Cas9 strategy used to knockout the second to fifth exon of Fbl in mouse macrophage cell line; **(B)** Verification of FBL deletion in *Fbl^+/-^* RAW264.7 cells by PCR followed by DNA gel electrophoresis; **(C)** Verification of FBL deletion in *Fbl^+/-^* RAW264.7 cells by Western blot; **(D)** Schematic diagram of the gene elements driving the expression of Cas9; **(E)** RT-qPCR of FBL sgRNA in WT (n=3) and *Fbl*-iKO RAW264.7 cells (n=5); (F) Western blot of FBL levels in WT and *Fbl*-iKO RAW264.7 cells. All data are mean ± SEM of biologically independent samples. ***P*<0.01, two-tailed unpaired Student's t test.

Supplementary Figure 5 RNA 2′-O-methyltransferase FBL does not regulate the expression of ISGs directly **(A)** qRT-PCR analysis of Ifi44, Oas2, Ifit3, Ddx58, Ddx60, Oasl1, Bst2, Ifit1 mRNA in mouse peritoneal macrophages transfected with the indicated siRNAs for 48 h (n=3), then treated with 500pg/ml IFNβ for 4 h; **(B)** qRT-PCR analysis of Ifi44, Oas2, Ifit3, Ddx58, Ddx60, Oasl1, Bst2, Ifit1 mRNA in WT and *Fbl^+/-^* RAW264.7 cells (n=9) stimulated with 500pg/ml IFNβ for 4 h. Data are mean ± SEM of biologically independent samples. ns, not significant, **P*<0.05, ***P*<0.01, *****P*<0.0001, two-tailed unpaired Student's t test.

Supplementary Table 1 Primers used for qRT-PCR.

Supplementary Table 2 siRNA sequences used for RNA interference.

Supplementary Table 3 Gradient elution procedure.

Supplementary Table 4 Methylated_sites in mRNA of RAW264.7 cells.

Supplementary Table 5 Up-regulated Nm sites after VSV infection.

Supplementary Table 6 mRNA Expression Profiling in *Fbl^+/+^* and *Fbl^+/-^* RAW264.7 cells.
