# Supplementary figures and images for: RNA 2’-O-Methyltransferase Fibrillarin Facilitates Virus Entry Into Macrophages Through Inhibiting Type I Interferon Response
Source: Front Immunol. 2022 Apr 7;13:793582. doi: 10.3389/fimmu.2022.793582 (PMC9021640; doi:10.3389/fimmu.2022.793582)

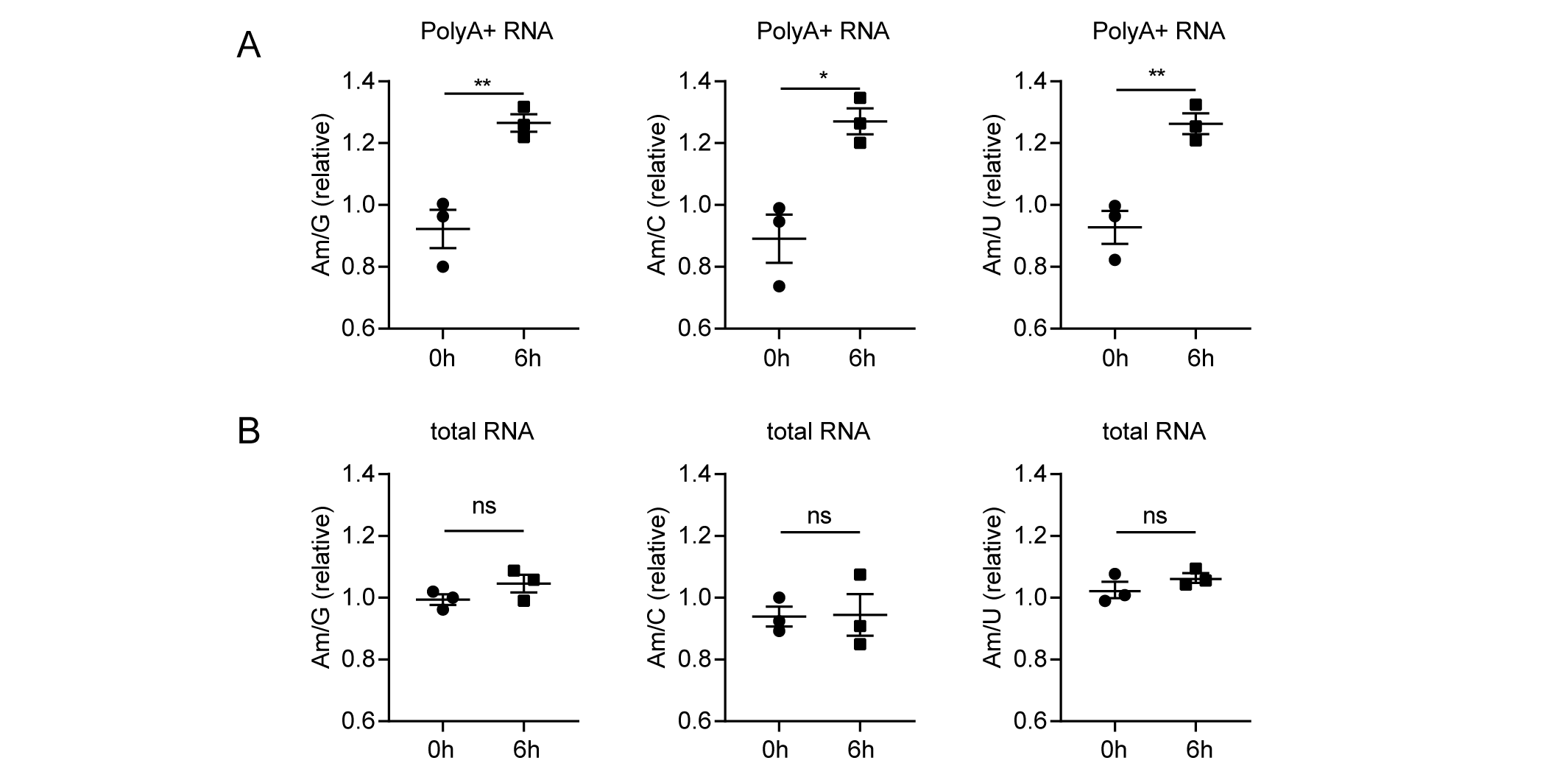

Supplement: Supplementary Figure 1 — Increased Am modification levls on poly A+ RNA in macrophages upon viral infection (A) Quantification of the Am/G, Am/C and Am/U ratio in poly A+ RNA of RAW264.7 cells with or without VSV infection (n=3); (B) Quantification of the Am/G, Am/C and Am/U ratio in total RNA of RAW264.7 cells with or without VSV infection (n=3). 0h, RNA from RAW264.7 cells; 6h, RNA from RAW264.7 cells infected with VSV (MOI=1) for 6 h. All data are mean ± SEM of biologically independent samples. ns, not significant, *P<0.05, **P<0.01, two-tailed unpaired Student's t test. [file Image_1.tif]

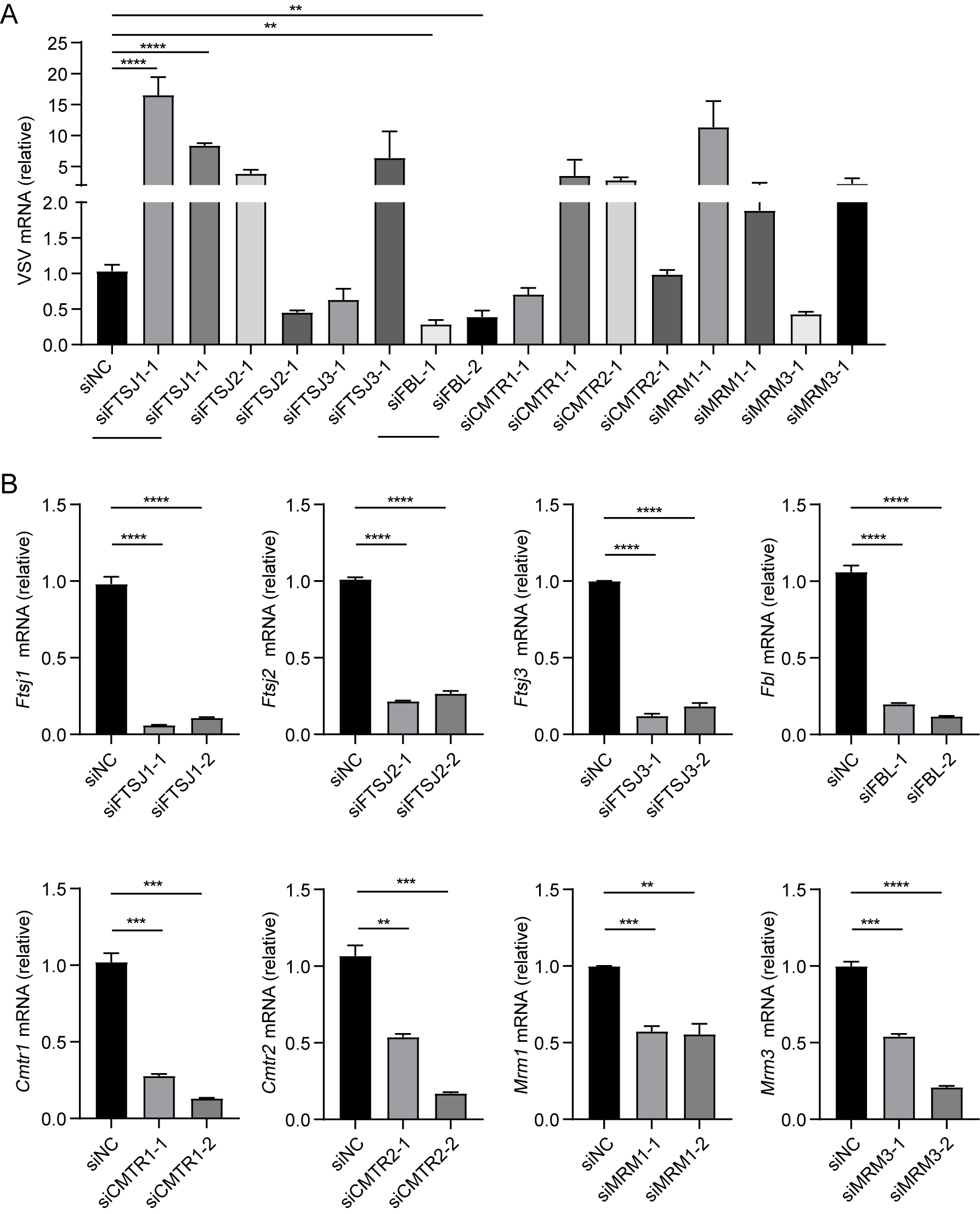

Supplement: Supplementary Figure 2 — RNAi screening of RNA 2′-O-methyltransferases in regulation of viral infection in macrophages (A) qRT-PCR of VSV RNA in mouse peritoneal macrophages transfected with the corresponding siRNA for 48h then infected with VSV (MOI=3) for 10 h (n=3). (B) qRT-PCR analysis mRNA expressions of Ftsj1, Ftsj2, Ftsj3, Fbl, Cmtr1, Cmtr2, Mrm1, Mrm3 in mouse peritoneal macrophages transfected with the corresponding siRNA for 48 h (n =3). All data are mean ± SEM of biologically independent samples. **P<0.01, ***P<0.001, ****P<0.0001, two-tailed unpaired Student’s t test. [file Image_2.tif]

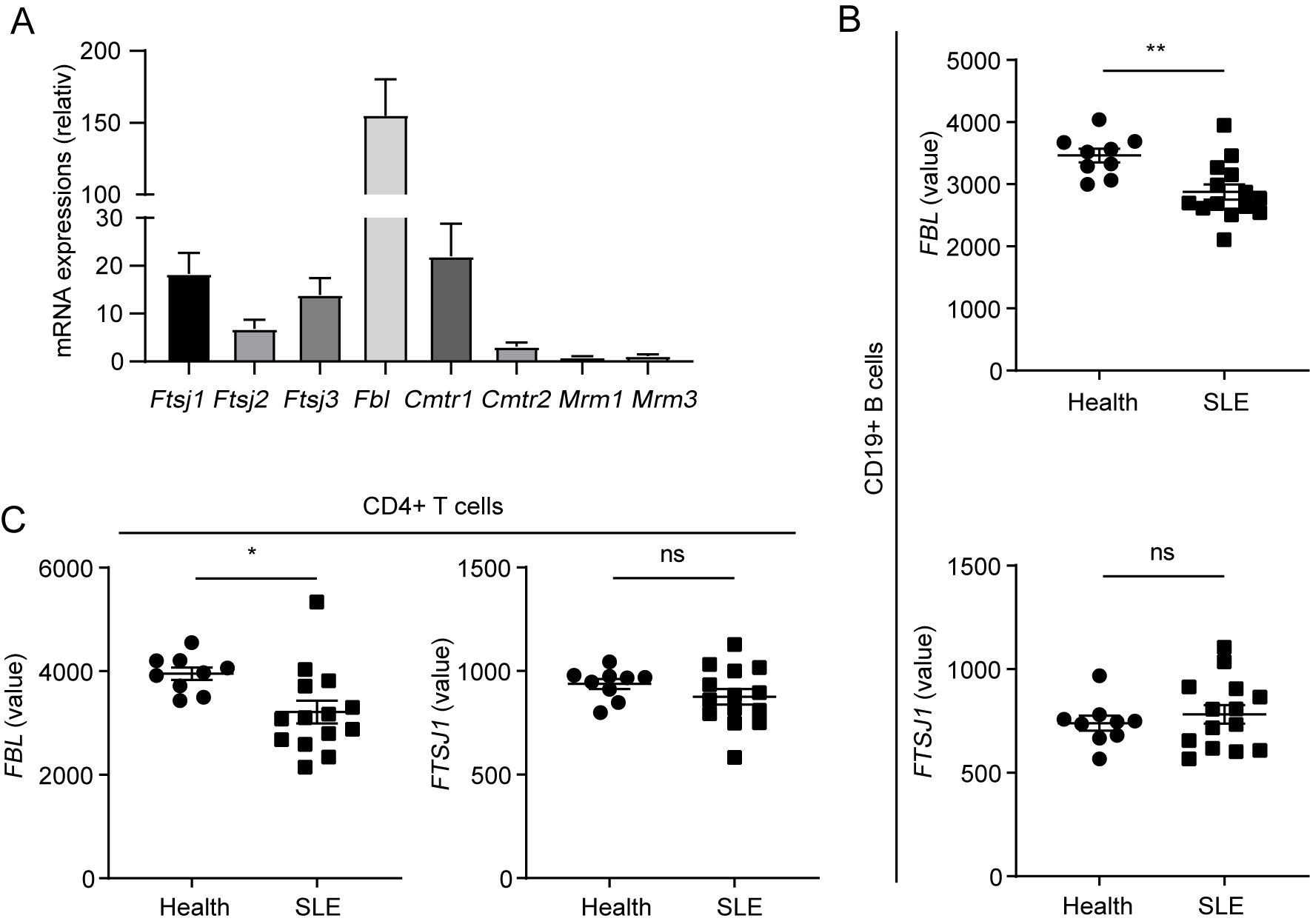

Supplement: Supplementary Figure 3 — Expression of FBL in mouse macrophages and human disease (A) qRT-PCR analysis mRNA expressions of Ftsj1, Ftsj2, Ftsj3, Fbl, Cmtr1, Cmtr2, Mrm1, Mrm3 in mouse peritoneal macrophages under steady state (n=3). Data are mean ± SEM of biologically independent samples; (B) FBL and FTSJ1 expression levels in CD19+ B cells of SLE patients (n=9) and healthy controls(n=14). Data are mean ± SEM resourced from GEO dataset GDS4185; (C) FBL and FTSJ1 expression levels in CD4+ T cells of SLE patients (n=9) and healthy controls (n=14). Data are mean ± SEM resourced from GEO dataset GDS4185. ns, not significant, *P<0.05, **P<0.01, two-tailed unpaired Student's t test. [file Image_3.tif]

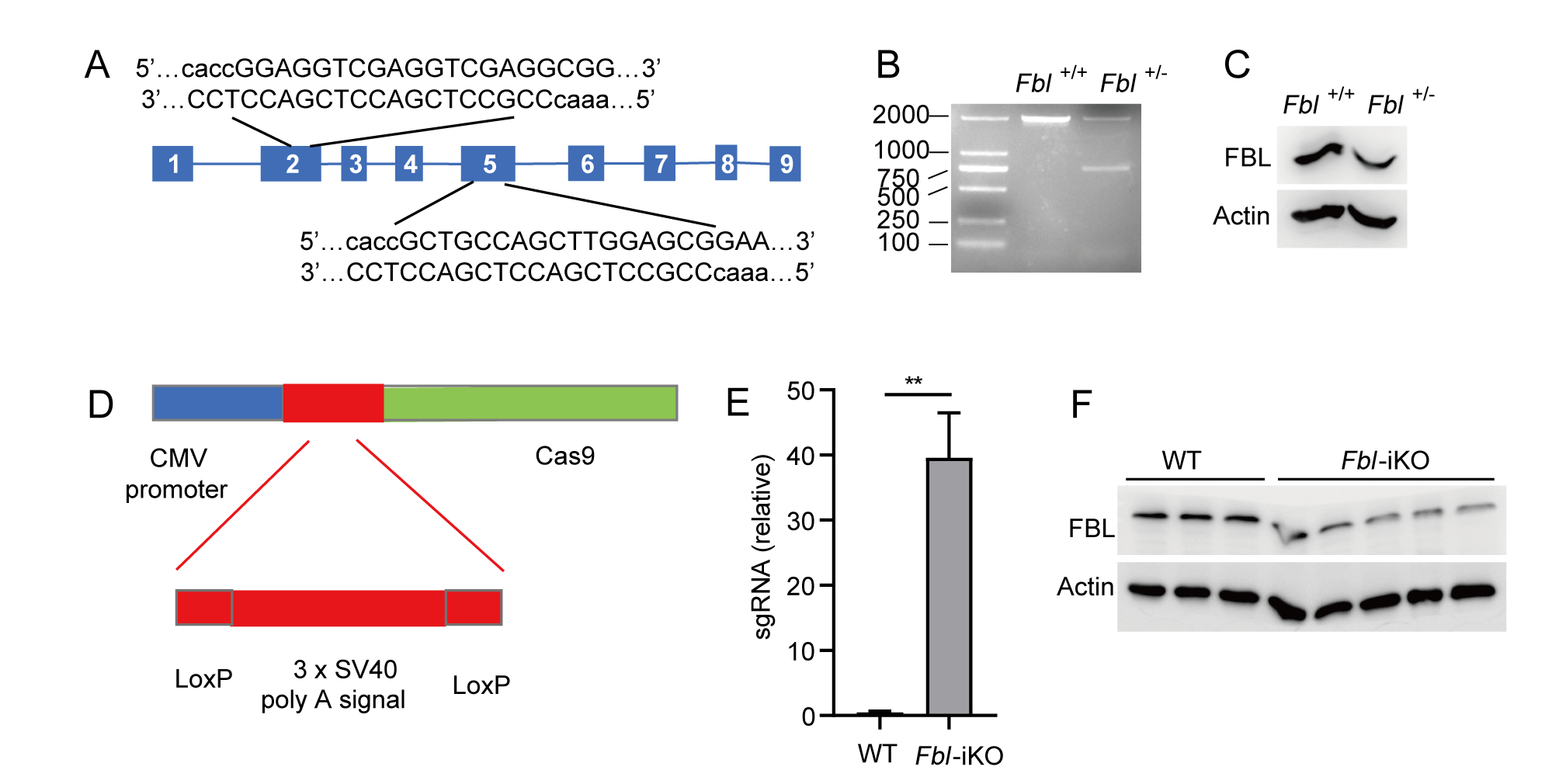

Supplement: Supplementary Figure 4 — RNA 2′-O-methyltransferase FBL facilitates viral infection (A) CRISPR/Cas9 strategy used to knockout the second to fifth exon of Fbl in mouse macrophage cell line; (B) Verification of FBL deletion in Fbl+/- RAW264.7 cells by PCR followed by DNA gel electrophoresis; (C) Verification of FBL deletion in Fbl+/- RAW264.7 cells by Western blot; (D) Schematic diagram of the gene elements driving the expression of Cas9; (E) RT-qPCR of FBL sgRNA in WT (n=3) and Fbl-iKO RAW264.7 cells (n=5); (F) Western blot of FBL levels in WT and Fbl-iKO RAW264.7 cells. All data are mean ± SEM of biologically independent samples. **P<0.01, two-tailed unpaired Student's t test. [file Image_4.tif]

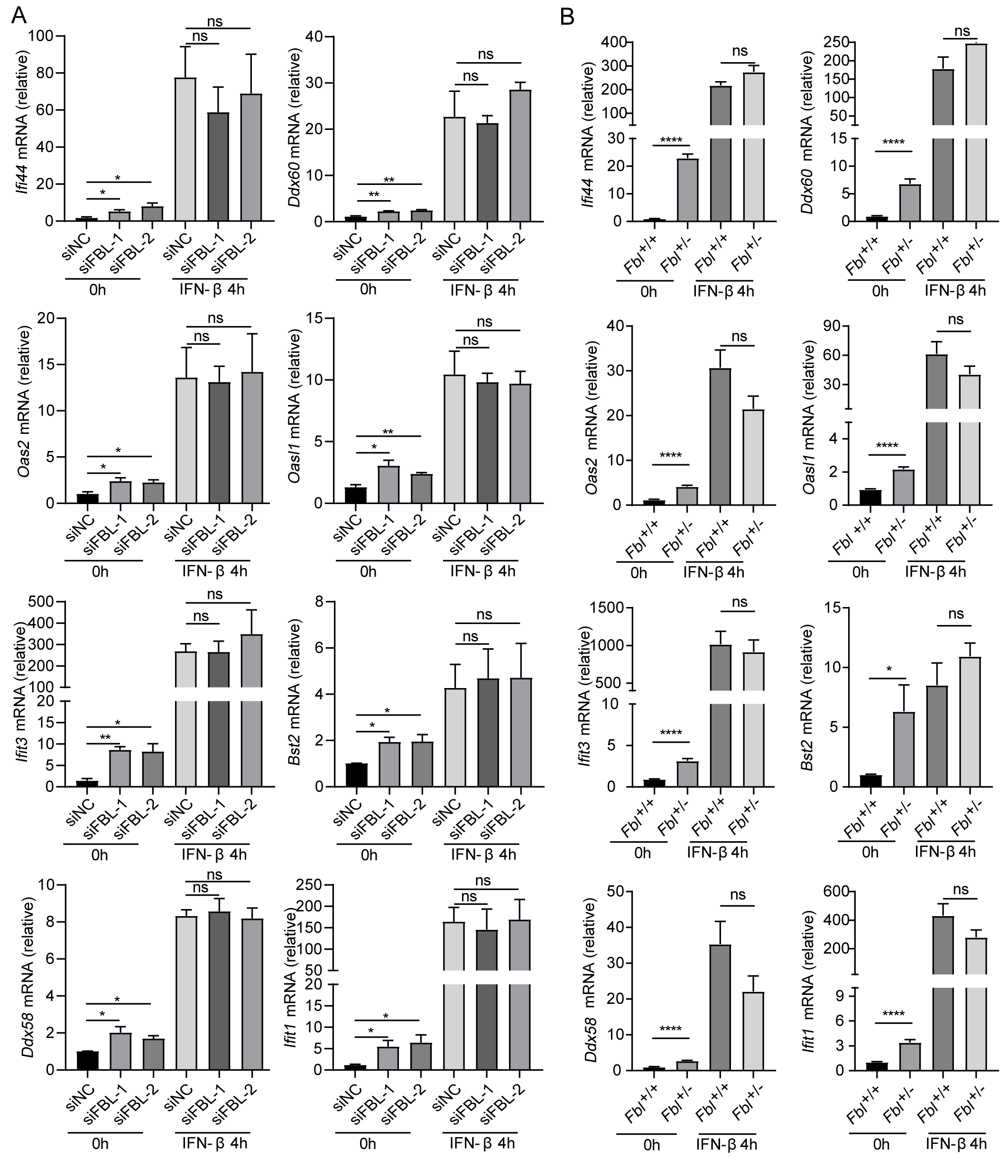

Supplement: Supplementary Figure 5 — RNA 2′-O-methyltransferase FBL does not regulate the expression of ISGs directly (A) qRT-PCR analysis of Ifi44, Oas2, Ifit3, Ddx58, Ddx60, Oasl1, Bst2, Ifit1 mRNA in mouse peritoneal macrophages transfected with the indicated siRNAs for 48 h (n=3), then treated with 500pg/ml IFNβ for 4 h; (B) qRT-PCR analysis of Ifi44, Oas2, Ifit3, Ddx58, Ddx60, Oasl1, Bst2, Ifit1 mRNA in WT and Fbl+/- RAW264.7 cells (n=9) stimulated with 500pg/ml IFNβ for 4 h. Data are mean ± SEM of biologically independent samples. ns, not significant, *P<0.05, **P<0.01, ****P<0.0001, two-tailed unpaired Student's t test. [file Image_5.tif]
