# Supplementary material for: RNA 2’-O-Methyltransferase Fibrillarin Facilitates Virus Entry Into Macrophages Through Inhibiting Type I Interferon Response
Source: Front Immunol. 2022 Apr 7;13:793582. doi: 10.3389/fimmu.2022.793582 (PMC9021640; doi:10.3389/fimmu.2022.793582)
Supplement: Supplementary Table 1 — Primers used for qRT-PCR. [file Table_1.docx]

**Supplementary Table 1. Primers used for qRT-PCR**

| Genes |  | Genes Primer sequences (5′ to 3′) |
| --- | --- | --- |
| Mouse *Ftsj1* | Forward | CTGCCCTGCTGACCTAGTAGT |
|  | Reverse | CAAGACATGAGTAGCGATGTTGA |
| Mouse *Ftsj2* | Forward | GAAGCTGGTAGGCGTTCCC |
|  | Reverse | CCTTTGCGGCCTTCACAAAC |
| Mouse *Ftsj3* | Forward | GGAGTGGACCTCGTTCCAATC |
|  | Reverse | GCGTCATGGACCCAACTAGC |
| Mouse *Fbl* | Forward | CAAAATTGAGTACAGAGCCTGGA |
|  | Reverse | CGGGCCGACAATATCAGAGA |
| Mouse *Cmtr1* | Forward | CTCCACATCTGACGATGAACC |
|  | Reverse | CTCCACAAGGGAATCTGCTTT |
| Mouse *Cmtr2* | Forward | CTGGTACTTCGGCCCAGATAA |
|  | Reverse | CACTAAGGCTTCCTGTTCACC |
| Mouse *Mrm3* | Forward | TAGCCGTCTGGAGTACGTCAA |
|  | Reverse | CGTTACTAGGTCTGACCAATCCT |
| Mouse *Mrm1* | Forward | CCCCATCACTAGCTGCTTAGA |
|  | Reverse | CGTCTGGGCAGAATAGTAAGGAG |
| Mouse *Actb* | Forward | AGTGTGACGTTGACATCCGT |
|  | Reverse | GCAGCTCAGTAACAGTCCGC |
| VSV | Forward | ACGGCGTACTTCCAGATGG |
|  | Reverse | CTCGGTTCAAGATCCAGGT |
| HSV-1 | Forward | ATACCGACGATCTGCGACCT |
|  | Reverse | TTATTGCCGTCATAGCGCGG |
| Mouse *Ifnb1* | Forward | CAGCTCCAAGAAAGGACGAAC |
|  | Reverse | GGCAGTGTAACTCTTCTGCAT |
| Mouse *Gapdh* | Forward | AGGTCGGTGTGAACGGATTTG |
|  | Reverse | TGTAGACCATGTAGTTGAGGTCA |
| Mouse *18s* | Forward | CAGCCACCCGAGATTGAGCA |
|  | Reverse | TAGTAGCGACGGGCGGGTGT |
| Mouse *Ifit1* | Forward | CTGAGATGTCACTTCACATGGAA |
|  | Reverse | GTGCATCCCCAATGGGTTCT |
| Mouse *Oas2* | Forward | TTGAAGAGGAATACATGCGGAAG |
|  | Reverse | GGGTCTGCATTACTGGCACTT |
| Mouse *Mx1* | Forward | GACCATAGGGGTCTTGACCAA |
|  | Reverse | AGACTTGCTCTTTCTGAAAAGCC |
| Mouse *Ifi44* | Forward | AACTGACTGCTCGCAATAATGT |
|  | Reverse | GTAACACAGCAATGCCTCTTGT |
| Mouse *Bst2* | Forward | TGTTCGGGGTTACCTTAGTCA |
|  | Reverse | GCAGGAGTTTGCCTGTGTCT |
| Mouse *Ifit3* | Forward | CCTACATAAAGCACCTAGATGGC |
|  | Reverse | ATGTGATAGTAGATCCAGGCGT |
| Mouse *Ddx60* | Forward | TTCCACTGCCCAAAATAGGAAAA |
|  | Reverse | GCCAGCAACATGAGTCTTAGGAT |
| Mouse *Oasl1* | Forward | CAGGAGCTGTACGGCTTCC |
|  | Reverse | CCTACCTTGAGTACCTTGAGCAC |
| Mouse *Rig-i* | Forward | ACAGATCCGAGACACTAAAGGG |
|  | Reverse | AACAGCGCCTCTGATGGAAAG |
| Human *ACTB* | Forward | AGAAGGATTCCTATGTGGGCG |
|  | Reverse | GGATAGCACAGCCTGGATAGCA |
| Human *IFNB1* | Forward | ACTGCAACCTTTCGAAGCCT |
|  | Reverse | AGCCTCCCATTCAATTGCCA |
| Human *IFNA4* | Forward | ATTTCTGCTCTGACAACCTC |
|  | Reverse | CTGAATGACTTGGAAGCCTG |
| Human *FBL* | Forward | CCTGGGGAATCAGTTTATGG |
|  | Reverse | CCAGGCTCGGTACTCAAT TT |
| Human *GAPDH* | Forward | CAACAGCCTCAAGATCATCA |
|  | Reverse | AGTCCTTCCACGATACCAA |
