# Supplementary material for: RNA 2’-O-Methyltransferase Fibrillarin Facilitates Virus Entry Into Macrophages Through Inhibiting Type I Interferon Response
Source: Front Immunol. 2022 Apr 7;13:793582. doi: 10.3389/fimmu.2022.793582 (PMC9021640; doi:10.3389/fimmu.2022.793582)
Supplement: Supplementary Table 2 — siRNA sequences used for RNA interference. [file Table_2.docx]

**Supplementary Table 2. siRNA sequences used for RNA interference**

| Genes |  | Genes Primer sequences (5′ to 3′) |
| --- | --- | --- |
| Mouse *Ftsj1* | 1 | GCCAACTACGTATCTTCTT |
|  | 2 | AGGAATTCCAACTCTTCAA |
| Mouse *Ftsj2* | 1 | CGATCTCCTTCACATATTC |
|  | 2 | AGAGACTGACCCAGGAATT |
| Mouse *Ftsj3* | 1 | CCAGCAGTTATTAGAGGAA |
|  | 2 | GGATGAAGATAGCTGGAAA |
| Mouse *Fbl* | 1 | CCTCCCAAGGTGAAGAACT |
|  | 2 | CGTCATGAAGGTGTCTTTA |
| Mouse *Cmtr1* | 1 | CTCTGTCAATCATGCAGCA |
|  | 2 | GAAGGAGTCGGACATTGA |
| Mouse *Cmtr2* | 1 | TCTGAGAACATTCGTCTTT |
|  | 2 | GTTCAGCCTCTGCTATCTA |
| Mouse *Mrm3* | 1 | CCAAACCTGACCCTGTTAA |
|  | 2 | AGAGGACCTAGACACTAAA |
| Mouse *Mrm1* | 1 | TCATCACCAGCCAGAGAAA |
|  | 2 | CCATCACTAGCTGCTTAGA |
| Human *FBL* | 1 | GGCCGTGACCTCATTAACT |
|  | 2 | GGCCGTGACCTCATTAACT |
