# Supplementary material for: RNA 2’-O-Methyltransferase Fibrillarin Facilitates Virus Entry Into Macrophages Through Inhibiting Type I Interferon Response
Source: Front Immunol. 2022 Apr 7;13:793582. doi: 10.3389/fimmu.2022.793582 (PMC9021640; doi:10.3389/fimmu.2022.793582)
Supplement: Supplementary Table 3 — Gradient elution procedure. [file Table_3.docx]

**Supplementary Table 3. Gradient elution procedure**

| Time(min^-1^) | A（%） | B（%） |
| --- | --- | --- |
| 0 | 100 | 0 |
| 6 | 100 | 0 |
| 7.65 | 99 | 1 |
| 9.35 | 94 | 6 |
| 10 | 94 | 6 |
| 12 | 50 | 50 |
| 14 | 25 | 75 |
| 17 | 25 | 75 |
| 17.5 | 100 | 0 |
| 38 | 100 | 0 |
